# The Association Between Low Back Pain and Composition of IgG Glycome

**DOI:** 10.1038/srep26815

**Published:** 2016-05-27

**Authors:** Maxim B. Freidin, Toma Keser, Ivan Gudelj, Jerko Štambuk, Dunja Vučenović, Massimo Allegri, Tamara Pavić, Mirna Šimurina, Stella M. Fabiane, Gordan Lauc, Frances M. K. Williams

**Affiliations:** 1 Department of Twin Research and Genetic Epidemiology, King’s College London, London, UK; 2 Department of Biochemistry and Molecular Biology, Faculty of Pharmacy and Biochemistry, University of Zagreb, Zagreb, Croatia; 3 Genos Glycoscience Research Laboratory, Zagreb, Croatia; 4 Department of Molecular Biology, Faculty of Science, University of Zagreb, Zagreb, Croatia; 5 Department of Surgical Science, University of Parma; Anaesthesia Intensive Care and Pain Therapy Service, Parma Hospital, Parma, Italy

## Abstract

Low back pain (LBP) is a common debilitating condition which aetiology and pathogenesis are poorly understood. We carried out a first so far analysis of associations between LBP and plasma IgG N-glycome in a sample of 4511 twins from TwinsUK database assessed for LBP, lumbar disc degeneration (LDD) as its possible cause, and IgG-glycan levels. Using weighted correlation network analysis, we established a correlation between LBP and glycan modules featured by glycans that either promote or block antibody-dependent cell-mediated cytotoxicity (ADCC). The levels of four glycan traits representing two of those modules were statistically significantly different in monozygotic twins discordant for LBP. Also, the trend to higher prevalence of systemic inflammatory disorders was shown for twins with low level of fucosylated glycans and high level of non-fucosylated glycans. Core fucosylation of IgG is a “safety switch” reducing ADCC, thus our results suggest the involvement of ADCC and associated inflammation in pathogenesis of LBP. No correlation between LDD scores and glycans was found assuming that the inflammation may not be a part of LDD. These data provide a new insight into understanding the complex pathophysiology of LBP and suggest glycan levels as a possible biomarker for inflammation-related subtypes of LBP.

Low back pain (LBP) is a common musculoskeletal condition in all ages^1^. The lifetime prevalence of non-specific LBP may reach 80%, with the annual prevalence ranging between 25% and 60% in different ethnic groups^2^,^3^. It is a diverse group of mixed pain syndromes with different molecular pathologies at different structural levels displaying similar clinical manifestations and radiologic findings. Why there is such huge inter-personal variability in severity of chronic LBP is yet to be clearly defined. Lumbar disk degeneration (LDD) is widely believed to be one of the major contributing factors. Nevertheless, MRI findings of disc degeneration cannot help to define clearly the pathophysiology of LBP and its prognosis^4^.

Even though, the development of LDD is associated with such occupational factors as heavy lifting, frequent bending and twisting^5^, genetic predisposition is much more important as a risk factor^6^.

A TwinsUK study showed genetic background as the major factor associated with LBP in women and also revealed a significant genetic correlation between LBP and LDD^7^. Genetic studies identified a dozen of genes associated with LDD, such as genes coding collagens, vitamin D receptor, interleukins, matrix metalloproteinases and other molecules^8^,^9^.

Large genome-wide linkage study and a genome-wide meta-analysis identified *CHST3* gene associated with LDD, and a subsequent functional analysis showed the risk allele decreases the gene expression, possibly, due to the enhanced interaction with miR-513a-5p microRNA^10^. Also, a meta-analysis of several genome-wide association studies revealed an association between LDD and *PARK* gene with the differential methylation of the *PARK* gene promoter as a possible cause for the association^11^. Also, an increased methylation of *SPARC* gene was found to be associated with LBP and LDD in humans and mice^12^. These studies incur epigenetic factors in the development of LDD and LBP.

Thus, the discovery of molecular factors contributing to the predisposition to LBP and LDD and mechanisms by which these factors act is essential to facilitate the development of new biomarkers of risk and response to specific treatments. Apart from genomic and epigenomic factors, other newly established “omes” can be of value.

In particular, glycome (the entire composition of glycans) attracts attention. Glycans constitute the most abundant and diverse form of the post-translational modifications. All cell surface and secreted glycoproteins that contain appropriate sequences (Asn-X-Ser/Thr where X is any amino acid except proline) can potentially acquire N-linked oligosaccharides (N-glycans) while they travel through the endoplasmic reticulum and the Golgi compartments. Glycans can influence disease development in many syndromes such as congenital disorders of glycosylation, cancer, rheumatoid arthritis and AIDS^13^. Glycans are crucial for the immune system, as some of the most important interactions between the immune system and viruses and bacteria are mediated by protein-glycan interactions. The biological functions of glycans go from basic structural roles to development, protein folding and immune response.

While genes unequivocally determine the structure of each polypeptide, there is no genetic template for the glycan part. Instead, hundreds of genes and their products interact in the complex pathway of glycan biosynthesis resulting in a very complex biosynthetic pathway that is further complicated by both direct environmental influence (nutrition, hormonal status, etc) and epigenetic memory of past environmental effects (altered gene expression)^14^,^15^,^16^. It is possible that some of the considerable genetic predisposition to LDD may be mediated via glycans.

Immunoglobulin G (IgG) glycosylation has been well defined, and many important functional effects of alternative IgG glycosylation have been described^17^. Glycans that lack terminal galactose activate complement and make IgG pro-inflammatory, while the addition of galactose decreases inflammatory potential of IgG^18^,^19^. Further extension of IgG glycans by the addition of sialic acid dramatically changes the physiological role of IgG, converting it from a pro-inflammatory into an anti-inflammatory agent. Terminal α2,6-sialylation of IgG glycans decreases the ability of IgG to bind to activating FcγRs and promotes recognition by DC-SIGN, which increases expression of inhibitory FcγRIIB and is anti-inflammatory^20^. Another example is the role of core fucose in the modulation of antibody-dependent cellular cytotoxicity: IgG-containing glycans that lack core fucose have 100-fold increased affinity for FcγRIIIA and are therefore much more efficient in activating antibody-dependent cellular cytotoxicity than fucosylated glycoforms of the same molecule^21^.

In this study, we analyzed twins from TwinsUK registry, to assess whether persons reporting episodes of LBP had detectable levels of altered IgG glycosylation.

## Results

The study aimed at identifying relationships between IgG glycosylation and pain. We used a cohort of twins from TwinsUK registry with established phenotypes of LBP and using a recently developed high-throughput analysis method quantified IgG glycans in their plasma specimens. After pre-processing and filtering of the data, a total of 4511 individuals were analyzed including 1215 pairs of DZ twins, 491 pairs of MZ twins, and 1099 unpaired individuals (Table 1). Low back pain status was known for 3557 individuals.

### Analysis of association between glycan levels and LBP

**Figure 1:**
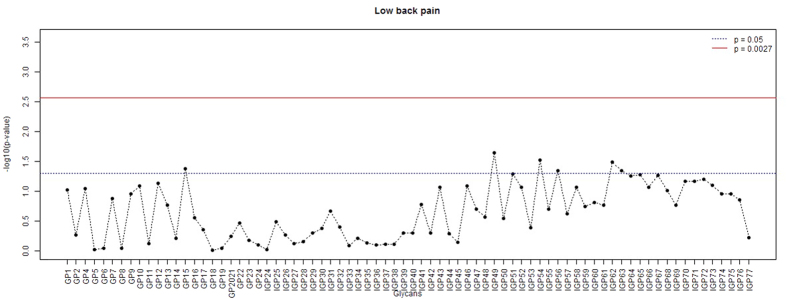
P-values (−log10) for the analysis of associations between glycan levels and low back pain. Linear mixed models were used to estimate the associations using LBP status, BMI, sex, and major inflammatory disease status as fixed factors and family status as a random factor.

Overall, 76 directly measured or derived glycan traits were assessed for an association with LBP (Supplementary Table 1). Linear mixed model analysis with BMI, sex, inflammatory diseases and LBP status included as fixed covariate and variation in IgG glycan quantities within twin pairs as random effect revealed nominally statistically significant associations of LBP with several glycan traits with the strongest association seen for IGP49 (GP10^n^) (Fig. 1). However, none of the associations passed the significance threshold set to control for multiple testing (p = 0.0027). Same was true for the analysis of correlations between glycan levels and summary scores for lumbar magnetic resonance imaging signs (LSUM; Supplementary Table 2; Supplementary Figure 1).

### WGCNA

Using the weighted correlation network analysis (WGCNA) methodology, we carried out a network analysis for glycan levels to establish clusters of correlated glycans which, possibly, reflect their functional relationships and revealed associations between these clusters and pain phenotypes.

**Figure 2:**
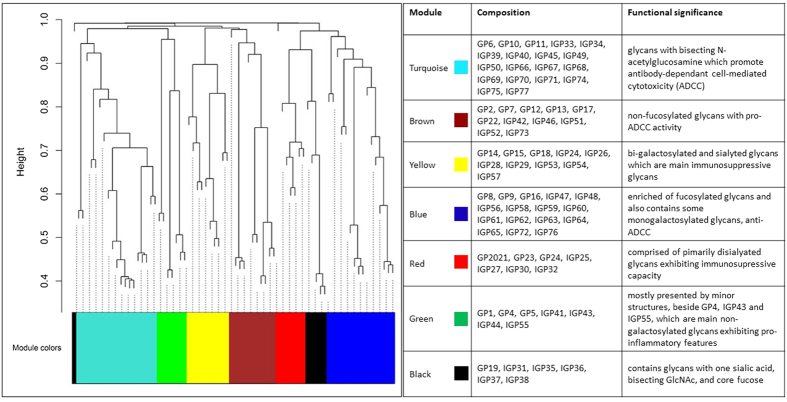
Modules of correlated glycans obtained using WGCNA methodology.

**Figure 3:**
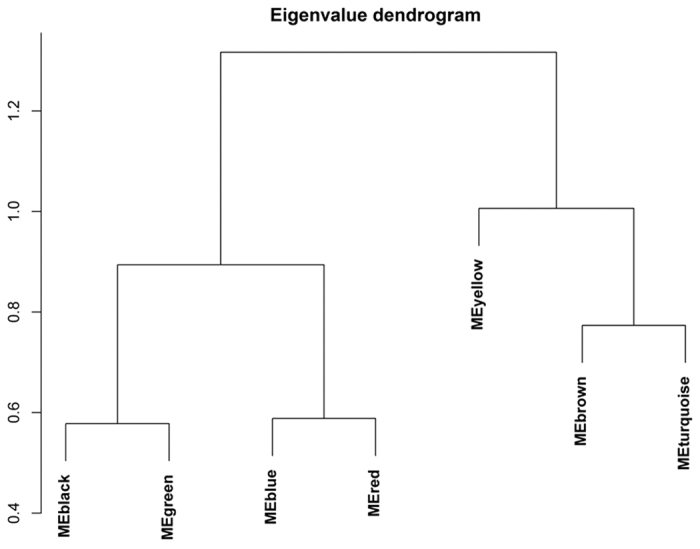
Relationships between modules of correlated glycans.

Using signed networks, we identified seven modules of correlated glycans (Fig. 2; Supplementary Table 1), which can be grouped into two big branches comprising yellow, brown and turquoise modules, from one hand, and black, green, blue and red modules, from the other hand (Fig. 3).

The most abundand turquoise module is comprised of glycans with bisecting *N*-acetylglucosamine (GlcNAc) which was reported to promote antibody-dependant cell-mediated cytotoxicity (ADCC)^22^. Similarly, the brown module belonging to the same branch of modules, contains non-fucosylated glycans, which also promote ADCC. The yellow module from the same branch is enriched of bi-galactosylated and sialylated glycans which are main immunosuppressive glycans.

Another branch’s biggest blue module, in opposition to the turquoise and brown modules, is enriched of fucosylated glycans, does not include glycans with bisecting GlcNAc, and also contains some monogalactosylated glycans. The red module is comprised of primarily disialyated glycans exhibiting immunosupressive capacity. The green module is mostly presented by minor structures, beside GP4, IGP43 (GP4^n^) and IGP55 (G0^n^), which are main non-galactosylated glycans exhibiting pro-inflammatory features. Finally, the black module contains glycans with one sialic acid, bisecting GlcNAc, and core fucose.

**Figure 4:**
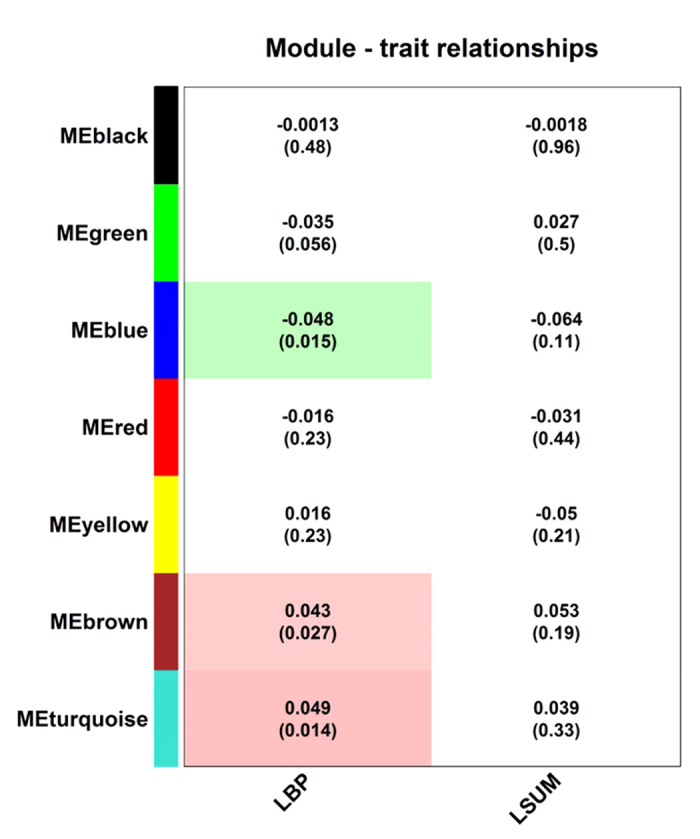
Correlations between module eigenvalues and pain phenotypes. Correlations were calculated between module eigenvalues (vector of first principal component of glycans in a module) and low back pain (LBP) using point-biserial correlation coefficient and summary score for magnetic resonance imaging signs for lumbar spine (LSUM) using Pearson correlation coefficients. Corresponding p-values are provided in brackets.

To reveal relationships between glycan modules and pain phenotypes, we carried out a correlation analysis between module eigenvalues (estimated as first principal component of glycan levels in a module) and LBP and MRI trait LSUM (Fig. 4). LBP was found to be positively correlated with turquoise and brown modules and negatively with blue module. A hint to correlation between green module and LBP was seen; however, these correlations did not reach statistical significance.

Also, even though the correlation strength between glycan modules and MRI-traits was of similar magnitude as those for LBP (R = 0.04–0.05), the correlations did not reach statistical significance for MRI-traits.

**Figure 5:**
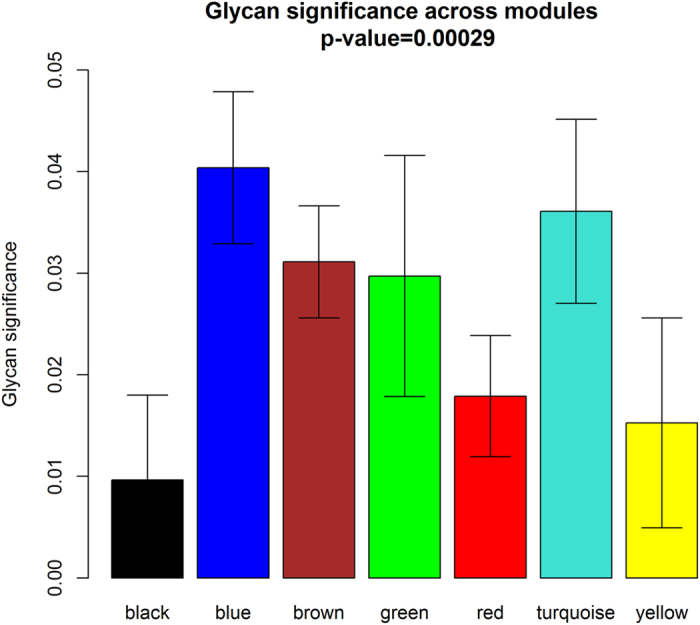
Average glycan significance across modules for LBP. Glycan significance was defined as the average coefficient of correlation between a trait and glycan levels in a module; p-value is given for Kruskal-Wallis test for the difference of glycan significance across the modules.

Average glycan significance for this phenotype (defined as the average for the correlation coefficients between glycan levels in a module and a trait) was highest for blue and turquoise modules for LBP (Fig. 5). These results suggest that glycans from the blue and turquoise modules may be of especial interest for subsequent study of their relationships with pain phenotype.

### Discordant twins analysis

To further analyse the relationships between glycome and pain phenotypes, we carried out comparisons of glycan levels in MZ and DZ twins discordant for LBP using paired t-test.

**Figure 6:**
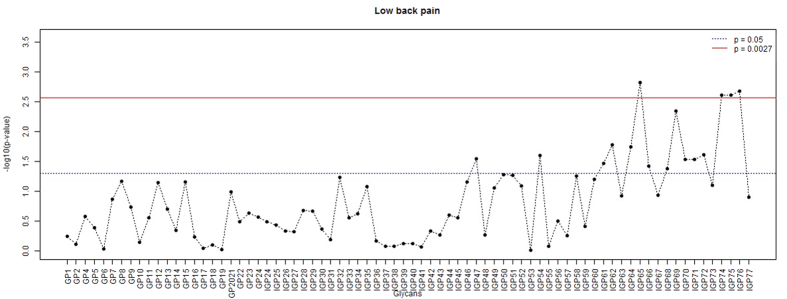
P-values (−log10) for comparisons of mean glycan levels in MZ twins discordant for LBP phenotype by paired t-test. Red line corresponds to p = 0.0027 which was taken as the significance threshold based on the 19 effective independent tests with Sidak’s correction for multiple testing.

**Figure 7:**
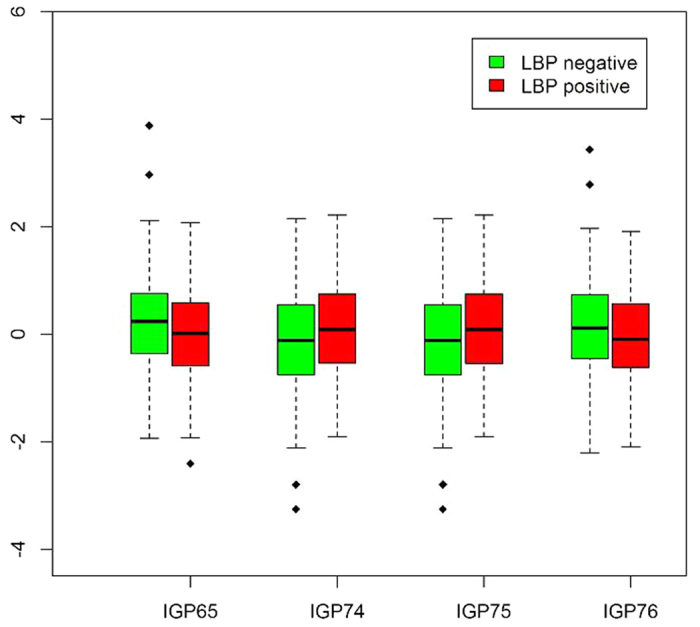
Glycan levels in MZ twins discordant for LBP phenotype.

For MZ twins, we identified statistically significant differences between the twins with and without LBP for the IGP65 (FG2^n^/G2^n^), IGP74 (FBG2^n^/FG2^n^), IGP75 (FBG2^n^ /[FG2^n^ + FBG2^n^ ]), and IGP76 (FG2^n^/[BG2^n^ + FBG2^n^]) derived traits (Fig. 6; p < 0.0027). Notably, these four glycan traits belong to the blue and turquoise modules identified in the WGCNA analysis. Accordingly, IGP65 and IGP76 of the blue module were found to be elevated in MZ twins without LBP, while IGP74 and IGP75 of the turquoise module were elevated in MZ twins with LBP (Fig. 7). The four glycan traits were derived from neutral glycans GP14 and GP15, and also GP13 for IGP76, with GP14 being the numerator for IGP65 and IGP76, while GP15 the numerator for the other two (Supplementary Table 1). Intriguingly, neither GP14, nor GP15 showed any trend to association with LBP; however, there was a weak, but significant negative correlation between GP14 and LCUM values (Pearson r = −0.08, p = 0.04; Supplementary Table 2; Supplementary Figure 1).

No statistical significant differences in glycan levels were found for DZ twins or MZ and DZ twins combined.

**Figure 8:**
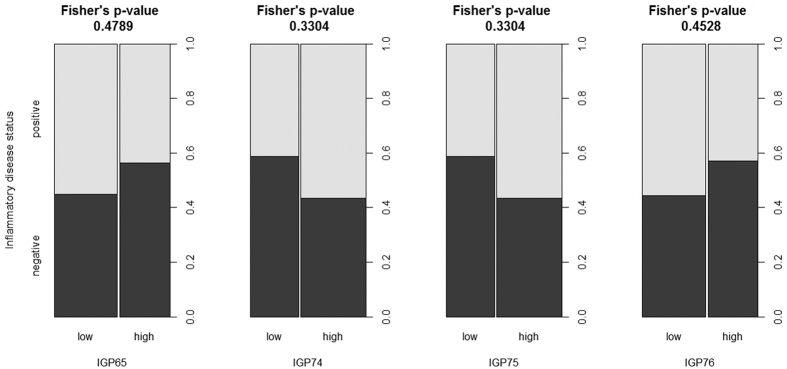
The prevalence of systemic inflammatory disorders (rheumatoid arthritis, systemic lupus erythematosus, ulcerative colitis, and Crohn’s disease) in twins with high and low levels of glycans and discordant for LBP. The cut off points for glycans levels were set at 25% and 75% quintile for the corresponding distribution.

To pursue a cause for association between LBP and glycan levels in MZ twins discordant for LBP, we split them into groups of high and low level of IGP65, IGP74, IGP75, and IGP76 using 25% and 75% quintiles as the cut off points and compared the prevalence of systemic inflammatory disorders (rheumatoid arthritis, systemic lupus erythematosus, ulcerative colitis and Crohn’s disease) in these groups. We found the increase of inflammatory diseases in individuals exhibiting low levels of IGP65 and IGP76 and high levels of IGP74 and IGP75 (Fig. 8). This pattern was in full agreement with the observation of association between these glycan levels and LBP, though the differences in inflammatory disorders prevalence did not reach statistical significance (according to Fisher’s exact test p-values).

## Discussion

### In this study we have evaluated association between levels of plasma IgG glycans and LBP

Linear mixed-models analysis did not reveal statistically significant (p < 0.0027) associations between glycan levels and LBP. However, for several glycans nominally significant associations were obtained.

In an attempt to consider glycome as a whole, we carried out a network analysis using weighted correlation network approach (WGCNA). This is a powerful methodology for revealing clusters (modules) of multiple omic traits, such as genome-wide gene expression or global methylation profiles, and placing them into a biological context through the analysis of associations between the clusters and diseases or traits of interest^23^,^24^,^25^,^26^,^27^,^28^. To the best of our knowledge, this method has never been applied before to glycome. Even though, glycans do not interact with each other in a way of genes or proteins, the network methodology underlying WGCNA analysis still seems valuable for glycome as it allows revealing functionally related groups of glycans exhibiting overlapping biological activity.

Using WGCNA approach we revealed seven modules of glycans clustered according to their functional capabilities, with the two biggest modules (turquoise and blue) enriched with glycans with opposite potential for the development of ADCC through the regulation of core fucosylation and bisection. Fucosylation is crucial in many biological processes and inflammation in particular. On average, 95% of the IgG population is core fucosylated^29^; core fucose prevents activation of ADCC, thus, most of the immunoglobulins have a “safety switch”, which prevents them from killing the target cell. Malfunction of this system appears to be associated with autoimmune diseases, as indicated by both pleiotropic effects of genes that associate with IgG glycosylation on different inflammatory and autoimmune diseases, and observed alterations in IgG glycosylation in systemic lupus erythematous^30^,^31^ and many inflammatory diseases^32^. We observed a positive correlation between LBP and “pro-ADCC” turquoise and brown modules and a negative correlation between LBP and “anti-ADCC” blue module (Fig. 4). Assuming that the development of LBP syndrome is in part related to inflammation^33^ and that ADCC contributes to the joint inflammation in some types of back pain^34^, one could expect that the decreased levels of core fucosylation and increased levels of bisecting GlcNAc in IgG glycans may contribute to increased ADCC and inflammation in LBP patiens. This observation corroborates with the finding of the significant difference in the levels of blue and turquoise module glycans in MZ twins discordant for LBP: IGP65 and IGP76 (blue module) were decreased in LBP-positive persons, while IGP74 and IGP75 (turquoise module) were increased in LBP-positive persons (Figs 6 and 7). Accordingly, we found the increased prevalence of major inflammatory diseases in LBP-discordant MZ twins with low levels of IGP65 and IGP76 and high levels of IGP75 and IGP76 (Fig. 8). Even though, the differences in the diseases prevalence were not statistically significant, the pattern of the differences corresponds to the pattern of association between the glycan levels and LBP, thus linking the three entities (glycans, LBP, and systemic inflammatory diseases). It is worth noting, that during inflammation process IgG glycome may vary in a quite complex way following several different patterns^35^. Therefore, the relationships between fucosylation, bisecting GlcNAc and LBP may not be entirely straightforward.

Interestingly, no statistically significant differences were found between DZ twins discordant for LBP. This may reflect a pronounced impact of environmental or gene-environment variability on co-variation between glycan levels and LBP.

## Conclusion

The current study was a first attempt to establish relationships between LBP and glycome. We proceeded from a hypothesis that LBP may be associated with an occult inflammation reflected by IgG glycan levels. We found consistent associations between pro- and anti-ADCC glycans with LBP, thus providing a proof for the tested hypothesis. Overall, our findings provide a further clue how inter-individual differences in IgG glycosylation might affect mechanisms of the development of LBP and suggest that glycans can be of interest as possible patient stratification biomarkers of this pain syndrome.

## Material and Methods

### Sample

Participants were a sample of MZ and DZ twins enlisted in the Twins UK registry^36^. The participants in the present study had undergone height and weight measurements used to calculate BMI. Collection of socio-demographic and LBP data was carried out during clinical visit or via a postal self-completion questionnaire. The twins were unaware of the precise research hypothesis addressed in the present study.

The study was carried out under the auspices of the FP7 PainOmics project and was approved by the St Thomas’ Hospital Research Ethics Committee. All the methods were carried out in accordance with the approved guidelines. All participating twins provided signed informed consent.

Participating twins underwent an assessment that included a nurse-led interview and a number of clinical and laboratory tests. As part of the study, the twins completed two standardized questionnaires relating to their lifetime history of low back symptoms. The questionnaires have been completed by each twin separately. The questionnaires included written questions and a mannequin pain diagram allowing an assessment of the timing, distribution, radiation, severity, and duration of pain together with information relating to functional disability. Low back pain was defined on a mannequin as being located between the 12th rib and the gluteal folds. First questionnaire followed the format of questions used in the UK Medical Research Council Nurses Study^37^ and the procedure of the assessment is detailed elsewhere^38^. The assessment of twins using this questionnaire was done in framework of the UK Twin Spine Study^7^,^39^. Another questionnaire followed the format of London Fibromyalgia Epidemiology Symptom Screening Questionnaire^40^. Specifically, the following questions have been used: “In the past month, have you had pain symptoms in this area (central lumbar region, left lumbar region, right lumbar region, left buttock, right buttock) lasting at least 24 hours? ” and “Have you had pain like this in this areas for at least the past 3 months? ”. This assessment using this questionnaire was done in framework of ongoing studies of chronic pain syndromes undertaken by the Department of Twin Research and Genetic Epidemiology at King’s College London^41^.

The LBP phenotype was defined as a binary trait based on questionnaire responses (1 = affected and 0 = nonaffected). Participants were categorised as cases for LBP if they reported having the syndrome with a total duration of >1 month and associated with disability according to the first questionnaire or at least 3 months according to the second questionnaire. Overall, 1656 and 2975 partly overlapping participants have been assessed using first and second questionnaire, respectively, which allowed identification of the LBP status in 3557 participants. Out of them, 585 participants (35.3% tested using first questionnaire) had disabling LBP lasting >1 month and 582 participants (20.0% tested using second questionnaire) had LBP lasting at least 3 months.

For 647 participants magnetic resonance imaging (MRI) was carried out as a part of LBP status assessment. The MRI scan was performed using a Siemens (Munich, Germany) 1.0 T superconducting magnet. Sagittal images were obtained using a fast spin-echo sequence of time to recovery (TR)/time to echo (TE) 5000–4500/112 msec, with a slice thickness of 4 mm. Grading was performed on T2-weighted images, although T1 images were also obtained for certain measurements. Axial sections were obtained at selected levels to assess structural changes in individuals who had features suggesting prolapse. To avoid problems related to diurnal variation in disc height all MRI scans were performed >1 hour after the subjects arose from sleep in the morning, with no exercise or other rest allowed between arising and the scan, and importantly, each twin pair was scanned at the same appointment and on the same machine^42^. A disease (LBP) severity score was constructed from the sum of scores for disc bulge, height, signal change, and narrowing in the lumbar spine (LSUM).

## Analysis of IgG glycans

### Isolation of IgG from Human Plasma

The IgG was isolated using protein G monolithic plates (BIA Separations, Ajdovščina, Slovenia) as described previously^29^. Briefly, 50 to 90 μL of serum was diluted 7 × with 1 × PBS, pH 7.4, applied to the protein G plate and instantly washed with 1 × PBS, pH 7.4, to remove unbound proteins. IgG was eluted with 1 mL of 0.1 M formic acid (Merck, Darmstadt, Germany) and neutralized with 1 M ammonium bicarbonate (Merck).

### Glycan Release and Labeling

IgG samples were first denatured with addition of 30 μL 1.33% sodium dodecyl sulfate (w/v) (Invitrogen, Carlsbad, CA) and by incubation at 65 °C for 10 min. Subsequently, 10 μL of 4% Igepal-CA630 (Sigma-Aldrich, St. Louis, MO) and 1.25 mU of PNGase F (ProZyme) in 10 μL 5 × phosphate-buffered saline were added to the samples. The samples were incubated overnight at 37 °C for N-glycan release. The released N-glycans were labeled with 2-AB. The labeling mixture was freshly prepared by dissolving 2-AB (Sigma–Aldrich) in dimethyl sulfoxide (Sigma–Aldrich) and glacial acetic acid (Merck) mixture (85:15, v/v) to a final concentration of 48 mg/mL. A volume of 25 μL of labeling mixture was added to each N-glycan sample in the 96-well plate. Also, 25 μL of freshly prepared reducing agent solution (106.96 mg/mL 2-picoline borane [Sigma-Aldrich] in dimethyl sulfoxide) was added and the plate was sealed using adhesive tape. Mixing was achieved by shaking for 10 min, followed by 2-hour incubation at 65 °C. Samples (in a volume of 100 μL) were brought to 80% acetonitrile (ACN) (v/v) by adding 400 μL of ACN (J.T. Baker, Phillipsburg, NJ). Free label and reducing agent were removed from the samples using hydrophilic interaction chromatography–solid-phase extraction. An amount of 200 μL of 0.1 g/mL suspension of microcrystalline cellulose (Merck) in water was applied to each well of a 0.45 μm GHP filter plate (Pall Corporation, Ann Arbor, MI). Solvent was removed by application of vacuum using a vacuum manifold (Millipore Corporation, Billerica, MA). All wells were prewashed using 5 × 200 μL water, followed by equilibration using 3 × 200 μL acetonitrile/water (80:20, v/v). The samples were loaded to the wells. The wells were subsequently washed seven times using 200 μL acetonitrile/water (80:20, v/v). Glycans were eluted two times with 100 μL of water and combined eluates were stored at −20 °C until usage.

### Hydrophilic Interaction Chromatography (HILIC)–Ultra Performance Liquid Chromatography

Fluorescently labeled N-glycans were separated by hydrophilic interaction chromatography on a Waters Acquity ultra performance liquid chromatography (UPLC) instrument (Milford, MA) consisting of a quaternary solvent manager, sample manager, and an FLR fluorescence detector set with excitation and emission wavelengths of 250 and 428 nm, respectively. The instrument was under the control of Empower 2 software, build 2145 (Waters). Labeled N-glycans were separated on a Waters bridged ethylene hybrid, glycan chromatography column, 100 × 2.1 mm internal diameter, 1.7-μm bridged ethylene hybrid particles, with 100 mM ammonium formate, pH 4.4, as solvent A and acetonitrile as solvent B. The separation method used a linear gradient of 75% to 62% acetonitrile (vol/vol) at flow rate of 0.4 mL/min in a 25-minute analytical run. Samples were maintained at 5 °C before injection, and the separation temperature was 60 °C. The system was calibrated using an external standard of hydrolyzed and 2-AB labeled glucose oligomers from which the retention times for the individual glycans were converted to glucose units. Data processing was performed using an automatic processing method with a traditional integration algorithm after which each chromatogram was manually corrected to maintain the same intervals of integration for all the samples. The chromatograms were all separated in the same manner into 24 peaks (GP1-GP24).

### Statistical analysis

#### Pre-processing and filtering

Directly measured glycan levels were normalized and experimental noise was removed through filtering and batch correction. Before this, we removed GP3 and combined GP20 and GP21 into a single trait.

First, we filtered out most extreme values from the dataset (beyond 0.999% percentile). Then, quotient normalization was applied using median values across the dataset as a reference^43^. Batch effect associated with different plates used to measure glycan levels was identified and corrected for using ratio-based method with either geometric mean or median^44^. As the results were almost equivalent, herewith, we report only the results for the dataset corrected with geometric mean.

After these steps, we estimated 55 derived glycan levels from the directly measured glycans^45^ using *glycanr* package for R [https://github.com/iugrina/glycanr] (Supplementary Table 1). These derived traits average particular glycosylation features (galactosylation, fucosylation, sialylation) across different individual glycan structures and consequently they are more closely related to individual enzymatic activities and underlying genetic polymorphisms. Finally, we applied inverse transformation of ranks to normality to obtain standard Normal distribution using *rntransform* function from *GenABEL* package for R^46^.

After pre-processing we assessed the dependency between the glycan traits and such confounders as age, sex, and body-mass index (BMI). For age piece-wise relationships with glycan levels were found, which made it unjustified adding age in linear regression models as a confounder. Therefore, before further analysis we corrected glycan levels for age (through residuals) by segmented regression using 40–45 years as initial break-down points as implemented in *segmented* package for R^47^. The choice of the break-down points was done based on the observation of the correlation clouds for age and glycans followed by a bootstrap based search for “true” breakpoints. Depending on specific glycans, both BMI and sex exhibited remarkable (and significant) to negligible (and insignificant) linear relationships with glycan levels.

#### Linear mixed-models analysis

Because of the twin structure of the dataset, association analyses between disease status and glycan traits were performed using linear mixed models with *lme4* package for R with BMI and sex included as fixed covariates and variation in IgG glycan quantities between twin pairs as random effect. Also, for 36 participants a diagnosis of a major systemic inflammatory disorder was established, including rheumatoid arthritis, systemic lupus erythematosus, ulcerative colitis, and Crohn’s disease. As people with such diagnoses normally undergo therapy with painkillers and anti-inflammatory medicine, and also the diseases have previously been found associated with variation in glycan levels, we included the diseases status as a fixed effect covariate in the analysis. The association was analysed for each glycan separately.

#### Weighted glycan “expression” networks

We used *WGCNA* package for R^48^,^49^ to carry out an exploratory analysis of “network” dependencies between the glycan traits. The algorithm of the analysis is based on the estimation of correlations between the glycan levels across the dataset followed by extraction of relatively independent modules of correlated glycans. Glycan levels were adjusted for age, sex, BMI, and inflammatory disease status before the analysis. Signed networks algorithm was used which takes into account the direction of the correlation between glycans. The modules (represented by their eigenvalue estimated as first principal component for the glycans in every module) then were correlated with the pain phenotypes, including LBP and MRI trait LSUM. To estimate correlations between glycan modules and pain phenotypes we used point-biserial correlation coefficients and Pearson’s correlation coefficients for qualitative and quantitative traits, respectively.

#### Discordant twins analysis

We compared the glycan levels in MZ and DZ twins discordant for LBP using paired t-test. Prior to the test, glycan levels were adjusted for age, sex, BMI, and inflammatory disease status.

#### The significance level consideration

There is an essential correlation between the glycan traits, many of which were derived from the original set of directly measured glycans. This complicates straightforward application of correction for multiple testing due to the violation of the requirement for the independence of the tests. Taking this into account, we estimated the effective number of independent statistical tests as of 19^50^, which after Sidak’s correction for multiple testing provided the significance level of 0.0027.

## Additional Information

**How to cite this article**: Freidin, M. B. *et al.* The Association Between Low Back Pain and Composition of lgG Glycome. *Sci. Rep.* **6**, 26815; doi: 10.1038/srep26815 (2016).

## Supplementary Material

Supplementary Information

## Figures and Tables

**Table 1:** Demographics of the studied twins.

Trait	Value	%
Age ± SD	51.8 ± 14.1	–
Females/males	4175/336	92.6/7.4
BMI ± SD	26.3 ± 5.0	–
LBP in total sample, positive/negative/unknown	1064/2493/954	23.6/55.3/21.1
LBP in pairs of twins, number of pairs		
MZ twins		
both positive/both negative/discordant/unknown in at least one of the twins	57/252/126/56	11.6/54.7/25.7/11.4
DZ twins		
both positive/both negative/discordant/unknown in at least one of the twins	146/452/316/301	12.0/37.2/26.0/24.8
