# Supplementary material for: The Association Between Low Back Pain and Composition of IgG Glycome
Source: Sci Rep. 2016 May 27;6:26815. doi: 10.1038/srep26815 (PMC4882546; doi:10.1038/srep26815)
Supplement: Supplementary Information [file srep26815-s1.pdf]

THE ASSOCIATION BETWEEN LOW BACK PAIN AND COMPOSITION OF IgG GLYCOME

Maxim B. Freidin[1,#], Toma Keser[2,#], Ivan Gudelj[3], Jerko Štambuk[3], Dunja Vučenović[1,4], Massimo Allegri[5], Tamara Pavić[2], Mirna Šimurina[2], Stella M. Fabiane[1], Gordan Lauc[2,3,$*], Frances M.K. Williams[1,$*]

Supplementary table 1. Structure and description of the studied IgG glycans

GROUP	Edinburgh Code	Zagreb Code	STRUCTURE[a]	DESCRIPTION	FORMULA
Total IgG glycans (neutral + charged)	IGP1	GP1	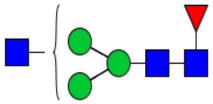	*The percentage of FA1 glycan in total IgG glycans*	*GP1 / GP* 100*
	IGP2	GP2	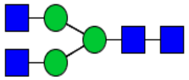	*The percentage of A2 glycan in total IgG glycans*	*GP2 / GP* 100*
	IGP3	GP4	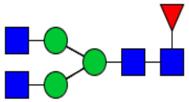	*The percentage of FA2 glycan in total IgG glycans*	*GP4 / GP* 100*
	IGP4	GP5	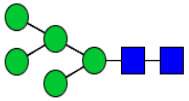	*The percentage of M5 glycan in total IgG glycans*	*GP5 / GP* 100*

	IGP5	GP6	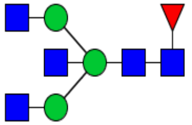	*The percentage of FA2B glycan in total IgG glycans*	*GP6 / GP* 100*
	IGP6	GP7	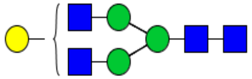	*The percentage of A2G1 glycan in total IgG glycans*	*GP7 / GP* 100*
	IGP7	GP8	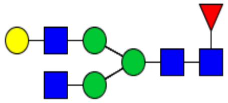	*The percentage of FA2[6]G1 glycan in total IgG glycans*	*GP8 / GP* 100*
	IGP8	GP9	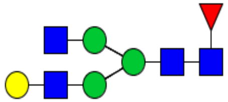	*The percentage of FA2[3]G1 glycan in total IgG glycans*	*GP9 / GP* 100*
	IGP9	GP10	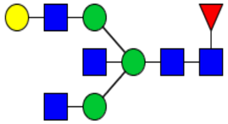	*The percentage of FA2[6]BG1 glycan in total IgG glycans*	*GP10 / GP* 100*
	IGP10	GP11	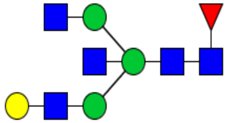	*The percentage of FA2[3]BG1 glycan in total IgG glycans*	*GP11 / GP* 100*
	IGP11	GP12	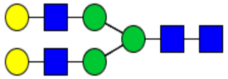	*The percentage of A2G2 glycan in total IgG glycans*	*GP12 / GP* 100*

	IGP12	GP13	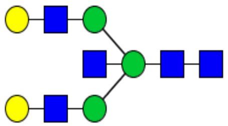	*The percentage of A2BG2 glycan in total IgG glycans*	*GP13 / GP* 100*
	IGP13	GP14	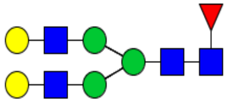	*The percentage of FA2G2 glycan in total IgG glycans*	*GP14 / GP* 100*
	IGP14	GP15	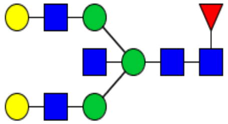	*The percentage of FA2BG2 glycan in total IgG glycans*	*GP15 / GP* 100*
	IGP15	GP16	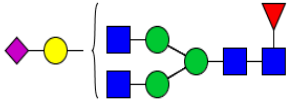	*The percentage of FA2G1S1 glycan in total IgG glycans*	*GP16 / GP * 100*
	IGP16	GP17	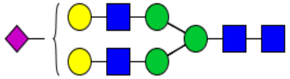	*The percentage of A2G2S1 glycan in total IgG glycans*	*GP17/ GP * 100*
	IGP17	GP18	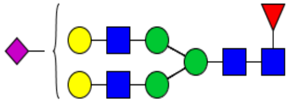	*The percentage of FA2G2S1 glycan in total IgG glycans*	*GP18 / GP * 100*

	IGP18	GP19	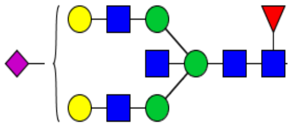	*The percentage of FA2BG2S1 glycan in total IgG glycans*	*GP19 / GP * 100*
	IGP19	GP20		*Structure not determined*	*GP20 / GP * 100*
	IGP20	GP21	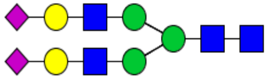	*The percentage of A2G2S2 glycan in total IgG glycans*	*GP21 / GP * 100*
	IGP21	GP22	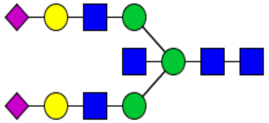	*The percentage of A2BG2S2 glycan in total IgG glycans*	*GP22 / GP * 100*
	IGP22	GP23	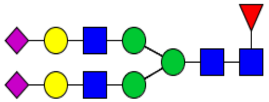	*The percentage of FA2G2S2 glycan in total IgG glycans*	*GP23 / GP * 100*
	IGP23	GP24	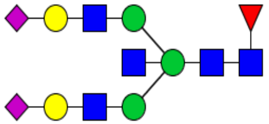	*The percentage of FA2BG2S2 glycan in total IgG glycans*	*GP24 / GP * 100*
Total IgG glycans - derived parameters	IGP24	FGS/(FG+FGS)		*The percentage of sialylation of fucosylated galactosylated structures without bisecting GlcNAc in total IgG glycans*	*SUM(GP16 + GP18 + GP23) / SUM(GP16 + GP18 + GP23 + GP8 + GP9 + GP14)* 100*
	IGP25	FBGS/(FBG+FBGS)		*The percentage of sialylation of fucosylated galactosylated structures with bisecting GlcNAc*	*SUM(GP19 + GP24) / SUM(GP19 + GP24 +*

				in total IgG glycans	*GP10 + GP11 + GP15)* 100*
	IGP26	FGS/(F+FG+FGS)		*The percentage of sialylation of all fucosylated structures without bisecting GlcNAc in total IgG glycans*	*SUM(GP16 + GP18 + GP23) / SUM(GP16 + GP18 + GP23 + GP4 + GP8 + GP9 + GP14)* 100*
	IGP27	FBGS/(FB+FBG+FBGS)		*The percentage of sialylation of all fucosylated structures with bisecting GlcNAc in total IgG glycans*	*SUM(GP19 + GP24) / SUM(GP19 + GP24 + GP6 + GP10 + GP11 + GP15)* 100*
	IGP28	FG1S1/(FG1+FG1S1)		*The percentage of monosialylation of fucosylated monogalactosylated structures in total IgG glycans*	*GP16 / SUM(GP16 + GP8 + GP9)* 100*
	IGP29	FG2S1/(FG2+FG2S1+FG2S2)		*The percentage of monosialylation of fucosylated digalactosylated structures in total IgG glycans*	*GP18 / SUM(GP18 + GP14 + GP23)* 100*
	IGP30	FG2S2/(FG2+FG2S1+FG2S2)		*The percentage of disialylation of fucosylated digalactosylated structures in total IgG glycans*	*GP23 / SUM(GP23 + GP14 + GP18)* 100*
	IGP31	FBG2S1/(FBG2+FBG2S1+FBG2S2)		*The percentage of monosialylation of fucosylated digalactosylated structures with bisecting GlcNAc in total IgG glycans*	*GP19 / SUM(GP19 + GP15 + GP24)* 100*
	IGP32	FBG2S2/(FBG2+FBG2S1+FBG2S2)		*The percentage of disialylation of fucosylated digalactosylated structures with bisecting GlcNAc in total IgG glycans*	*GP24 / SUM(GP24 + GP15 + GP19)* 100*
	IGP33	$F^{total}S1/F^{total}S2$		*Ratio of all fucosylated (+/- bisecting GlyNAc) monosyalilated and disialylated structures in total IgG glycans*	*SUM(GP16 + GP18 + GP19) / SUM(GP23 + GP24)*
	IGP34	FS1/FS2		*Ratio of fucosylated (without bisecting GlcNAc) monosialylated and disialylated structures in total IgG glycans*	*SUM(GP16 + GP18) / GP23*
	IGP35	FBS1/FBS2		*Ratio of fucosylated (with bisecting GlcNAc) monosialylated and disialylated structures in total IgG glycans*	*GP19 / GP24*
	IGP36	FBS^{total}/FS^{total}		*Ratio of all fucosylated sialylated structures*	*SUM(GP19 + GP24) /*

				with and without bisecting GlcNAc	*SUM(GP16 + GP18 + GP23)*
	IGP37	FBS1/FS1		*Ratio of fucosylated monosialylated structures with and without bisecting GlcNAc*	*GP19 / SUM(GP16 + GP18)*
	IGP38	FBS1/(FS1+FBS1)		*The incidence of bisecting GlcNAc in all fucosylated monosialylated structures in total IgG glycans*	*GP19 / SUM(GP16 + GP18 + GP19)*
	IGP39	FBS2/FS2		*Ratio of fucosylated disialylated structures with and without bisecting GlcNAc*	*GP24 / GP23*
	IGP40	FBS2/(FS2+FBS2)		*The incidence of bisecting GlcNAc in all fucosylated disialylated structures in total IgG glycans*	*GP24 / SUM(GP23 + GP24)*
Neutral IgG glycans	IGP41	$GP1^n$		*The percentage of FA1 glycan in total neutral IgG glycans (GP^n)*	*GP1 / GP^n * 100*
	IGP42	$GP2^n$		*The percentage of A2 glycan in total neutral IgG glycans (GP^n)*	*GP2 / GP^n * 100*
	IGP43	$GP4^n$		*The percentage of FA2 glycan in total neutral IgG glycans (GP^n)*	*GP4 / GP^n * 100*
	IGP44	$GP5^n$		*The percentage of M5 glycan in total neutral IgG glycans (GP^n)*	*GP5 / GP^n * 100*
	IGP45	$GP6^n$		*The percentage of FA2B glycan in total neutral IgG glycans (GP^n)*	*GP6 / GP^n * 100*
	IGP46	$GP7^n$		*The percentage of A2G1 glycan in total Ineutral IgG glycans (GP^n)*	*GP7 / GP^n * 100*
	IGP47	$GP8^n$		*The percentage of FA2[6]G1 glycan in total neutral IgG glycans (GP^n)*	*GP8 / GP^n * 100*
	IGP48	$GP9^n$		*The percentage of FA2[3]G1 glycan in total neutral IgG glycans (GP^n)*	*GP9 / GP^n * 100*
	IGP49	$GP10^n$		*The percentage of FA2[6]BG1 glycan in total neutral IgG glycans (GP^n)*	*GP10 / GP^n * 100*
	IGP50	$GP11^n$		*The percentage of FA2[3]BG1 glycan in total neutral IgG glycans (GP^n)*	*GP11 / GP^n * 100*
	IGP51	$GP12^n$		*The percentage of A2G2 glycan in total neutral IgG glycans (GP^n)*	*GP12 / GP^n * 100*
	IGP52	$GP13^n$		*The percentage of A2BG2 glycan in total*	*GP13 / GP^n * 100*

				neutral IgG glycans (GP^n)	
	IGP53	$GP14^n$		*The percentage of FA2G2 glycan in total neutral IgG glycans (GP^n)*	*$GP14 / GP^n * 100$*
	IGP54	$GP15^n$		*The percentage of FA2BG2 glycan in total neutral IgG glycans (GP^n)*	*$GP15 / GP^n * 100$*
Neutral IgG glycans - derived parameters	IGP55	$G0^n$		*The percentage of agalactosylated structures in total neutral IgG glycans*	*SUM($GP1^n$: $GP6^n$)*
	IGP56	$G1^n$		*The percentage of monogalactosylated structures in total neutral IgG glycans*	*SUM($GP7^n$: $GP11^n$)*
	IGP57	$G2^n$		*The percentage of digalactosylated structures in total neutral IgG glycans*	*SUM($GP12^n$: $GP15^n$)*
	IGP58	$F^{n\ total}$		*The percentage of all fucosylated (+/- bisecting GlcNAc) structures in total neutral IgG glycans*	*SUM($GP1^n$+ $GP4^n$+ $GP5^n$+ $GP6^n$+ $GP8^n$+ $GP9^n$+ $GP10^n$+ $GP11^n$+ $GP14^n$+ $GP15^n$)*
	IGP59	$FG0^{n\ total}/G0^n$		*The percentage of fucosylation of agalactosylated structures*	*SUM($GP1^n$+ $GP4^n$+ $GP5^n$+ $GP6^n$) / $G0^n$ * 100*
	IGP60	$FG1^{n\ total}/G1^n$		*The percentage of fucosylation of monogalactosylated structures*	*SUM($GP8^n$+ $GP9^n$+ $GP10^n$+ $GP11^n$) / $G1^n$ * 100*
	IGP61	$FG2^{n\ total}/G2^n$		*The percentage of fucosylation of digalactosylated structures*	*SUM($GP14^n$+ GP15) / $G2^n$ * 100*
	IGP62	F^n		*The percentage of fucosylated (without bisecting GlcNAc) structures in total neutral IgG glycans*	*SUM($GP1^n$+ $GP4^n$+ $GP5^n$+ $GP8^n$+ $GP9^n$+ $GP14^n$)*
	IGP63	$FG0^n/G0^n$		*The percentage of fucosylation (without bisecting GlcNAc) of agalactosylated structures*	*SUM($GP1^n$+ $GP4^n$+ $GP5^n$) / $G0^n$ * 100*
	IGP64	$FG1^n/G1^n$		*The percentage of fucosylation (without bisecting GlcNAc) of monogalactosylated structures*	*SUM($GP8^n$+ $GP9^n$) / $G1^n$ * 100*
	IGP65	$FG2^n/G2^n$		*The percentage of fucosylation (without bisecting GlcNAc) of digalactosylated structures*	*$GP14^n$/ $G2^n$ * 100*
	IGP66	FB^n		*The percentage of fucosylated (with bisecting GlcNAc) structures in total neutral IgG glycans*	*SUM($GP6^n$ + $GP10^n$ + $GP11^n$ + $GP15^n$)*

	IGP67	FBG0^{n}/G0^{n}		*The percentage of fucosylation (with bisecting GlcNAc) of agalactosylated structures*	*GP6^{n}/ G0^{n} * 100*
	IGP68	FBG1^{n}/G1^{n}		*The percentage of fucosylation (with bisecting GlcNAc) of monogalactosylated structures*	*SUM(GP10^{n} + GP11^{n}) / G1^{n} * 100*
	IGP69	FBG2^{n}/G2^{n}		*The percentage of fucosylation (with bisecting GlcNAc) of digalactosylated structures*	*GP15) / G2^{n} * 100*
	IGP70	FB^{n}/F^{n}		*Ratio of fucosylated structures with and without bisecting GlcNAc*	*FB^{n}/ F^{n} * 100*
	IGP71	FB^{n}/F$^{n\ total}$		*The incidence of bisecting GlcNAc in all fucosylated structures in total neutral IgG glycans*	*FB^{n}/ F$^{n\ total}$ * 100*
	IGP72	F^{n}/(B^{n} + FB^{n})		*Ratio of fucosylated non-bisecting GlcNAc structures and all structures with bisecting GlcNAc*	*F^{n}/(GP13^{n} + FB^{n})*
	IGP73	B^{n}/(F^{n} + FB^{n})		*Ratio of structures with bisecting GlcNAc and all fucosylated structures (+/- bisecting GlcNAc)*	*GP13^{n}/ (F^{n}+ FB^{n}) * 1000*
	IGP74	FBG2^{n}/FG2^{n}		*Ratio of fucosylated digalactosylated structures with and without bisecting GlcNAc*	*GP15^{n}/GP14^{n}*
	IGP75	FBG2^{n} /(FG2^{n} + FBG2^{n})		*The incidence of bisecting GlcNAc in all fucosylated digalactosylated structures in total neutral IgG glycans*	*GP15^{n}/(GP14^{n} + GP15^{n}) * 100*
	IGP76	FG2^{n}/(BG2^{n} + FBG2^{n})		*Ratio of fucosylated digalactosylated non-bisecting GlcNAc structures and all digalactosylated structures with bisecting GlcNAc*	*GP14^{n}/(GP13^{n} + GP15^{n})*
	IGP77	BG2^{n}/(FG2^{n} + FBG2^{n})		*Ratio of digalactosylated structures with bisecting GlcNAc and all fucosylated digalactosylated structures (+/- bisecting GlcNAc)*	*GP15^{n}/(GP14^{n} + GP15^{n}) * 1000*

[a]Symbol nomenclature for glycan representation was used according to: Varki A, Cummings RD, Esko JD, et al. Symbol nomenclature for glycan representation. Proteomics. 2009;9(24):5398-5399. doi:10.1002/pmic.200900708.

Supplementary Table 2. Correlations between glycan levels and summary scores for lumbar magnetic resonance imaging signs

Glycan	Correlation coefficient*	p-value
**GP1**	-0.008	0.835
**GP2**	0.085	0.032
**GP4**	-0.001	0.974
**GP5**	0.063	0.115
**GP6**	0.024	0.549
**GP7**	0.066	0.096
**GP8**	-0.014	0.725
**GP9**	-0.045	0.255
**GP10**	0.005	0.907
**GP11**	0.011	0.781
**GP12**	0.022	0.574
**GP13**	-0.011	0.781
**GP14**	-0.083	0.037
**GP15**	-0.044	0.265
**GP16**	-0.016	0.681
**GP17**	0.041	0.306
**GP18**	-0.064	0.109
**GP19**	-0.043	0.281
**GP2021**	0.045	0.258
**GP22**	-0.011	0.783
**GP23**	-0.050	0.211
**GP24**	-0.053	0.183
**IGP24**	-0.021	0.606
**IGP25**	-0.031	0.433
**IGP26**	-0.029	0.462
**IGP27**	-0.043	0.281
**IGP28**	0.012	0.755
**IGP29**	0.045	0.260
**IGP30**	0.014	0.720
**IGP31**	0.003	0.938
**IGP32**	-0.017	0.670
**IGP33**	0.002	0.962
**IGP34**	0.004	0.917
**IGP35**	0.009	0.831
**IGP36**	0.009	0.819
**IGP37**	0.008	0.846
**IGP38**	0.008	0.847
**IGP39**	0.019	0.634
**IGP40**	0.019	0.634
**IGP41**	0.001	0.976
**IGP42**	0.095	0.017

**IGP43**	0.020	0.623
**IGP44**	0.082	0.040
**IGP45**	0.035	0.381
**IGP46**	0.069	0.082
**IGP47**	0.007	0.864
**IGP48**	-0.035	0.378
**IGP49**	0.023	0.569
**IGP50**	0.030	0.459
**IGP51**	0.027	0.491
**IGP52**	0.004	0.917
**IGP53**	-0.063	0.115
**IGP54**	-0.021	0.607
**IGP55**	0.031	0.442
**IGP56**	0.014	0.718
**IGP57**	-0.053	0.183
**IGP58**	-0.061	0.126
**IGP59**	-0.086	0.030
**IGP60**	-0.067	0.091
**IGP61**	-0.070	0.081
**IGP62**	-0.050	0.211
**IGP63**	-0.043	0.285
**IGP64**	-0.035	0.383
**IGP65**	-0.081	0.042
**IGP66**	0.033	0.406
**IGP67**	0.023	0.560
**IGP68**	0.021	0.594
**IGP69**	0.056	0.162
**IGP70**	0.038	0.341
**IGP71**	0.038	0.341
**IGP72**	-0.038	0.342
**IGP73**	0.007	0.851
**IGP74**	0.064	0.111
**IGP75**	0.064	0.111
**IGP76**	-0.068	0.087
**IGP77**	0.072	0.072

* Pearson's correlations were estimated after adjusting glycan levels for age, sex, BMI, and inflammatory disease status.

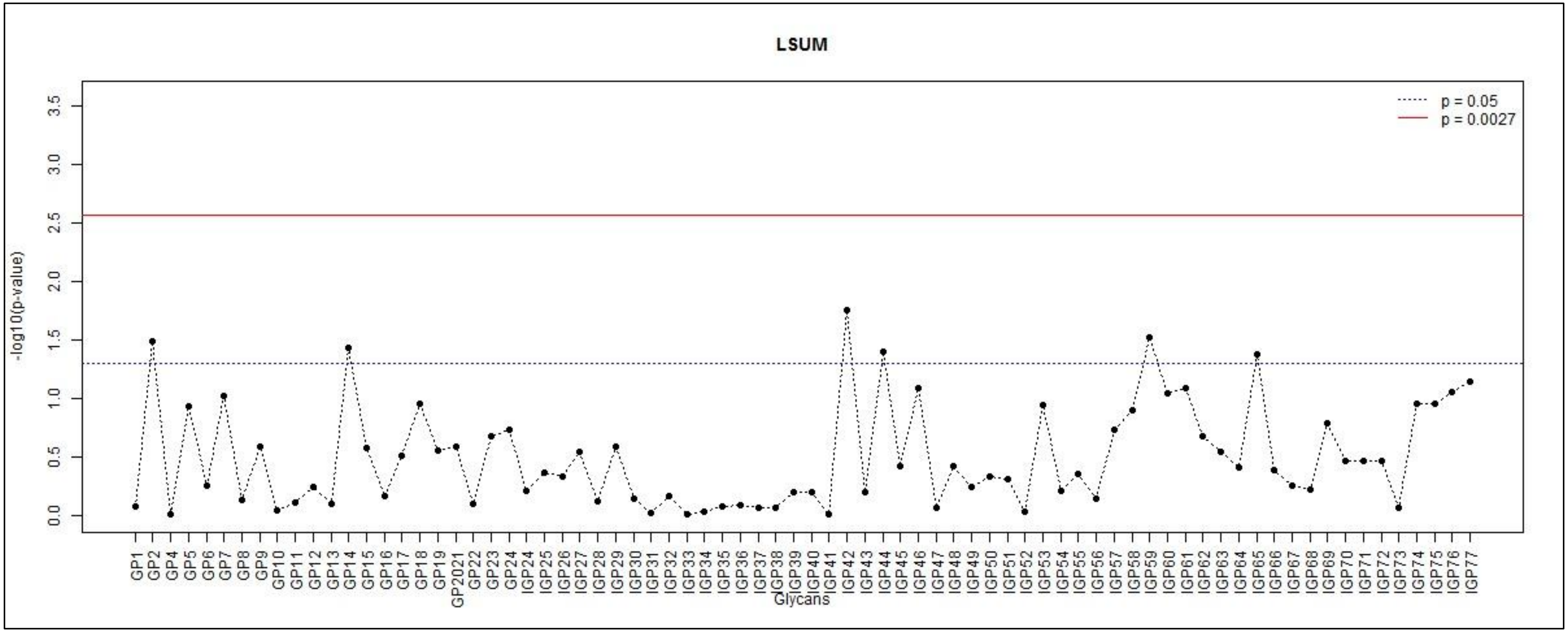

Supplementary Figure 1. P-values (-log10) for the analysis of correlations between glycan levels and LSUM MRI scores.

P-values correspond to Pearson's correlation coefficients provided in Supplementary table 2.
